# Identification of scavenger receptors and thrombospondin‐type‐1 repeat proteins potentially relevant for plastid recognition in Sacoglossa

**DOI:** 10.1002/ece3.6865

**Published:** 2020-10-12

**Authors:** Jenny Melo Clavijo, Silja Frankenbach, Cátia Fidalgo, João Serôdio, Alexander Donath, Angelika Preisfeld, Gregor Christa

**Affiliations:** ^1^ Fakultät für Mathematik und Naturwissenschaften, Zoologie und Biologiedidaktik Bergische Universität Wuppertal Wuppertal Germany; ^2^ Department of Biology and CESAM – Center for Environmental and Marine Studies University of Aveiro Aveiro Portugal; ^3^ Center for Molecular Biodiversity Research Zoological Research Museum Alexander Koenig Bonn Germany

**Keywords:** *Elysia*, Kleptoplasty, photosymbiosis, sacoglossa, scavenger receptors, thrombospondin

## Abstract

Functional kleptoplasty is a photosymbiotic relationship, in which photosynthetically active chloroplasts serve as an intracellular symbiont for a heterotrophic host. Among Metazoa, functional kleptoplasty is only found in marine sea slugs belonging to the Sacoglossa and recently described in Rhabdocoela worms. Although functional kleptoplasty has been intensively studied in Sacoglossa, the fundamentals of the specific recognition of the chloroplasts and their subsequent incorporation are unknown. The key to ensure the initiation of any symbiosis is the ability to specifically recognize the symbiont and to differentiate a symbiont from a pathogen. For instance, in photosymbiotic cnidarians, several studies have shown that the host innate immune system, in particular scavenger receptors (SRs) and thrombospondin‐type‐1 repeat (TSR) protein superfamily, is playing a major role in the process of recognizing and differentiating symbionts from pathogens. In the present study, SRs and TSRs of three Sacoglossa sea slugs, *Elysia cornigera*, *Elysia timida*, and *Elysia chlorotica*, were identified by translating available transcriptomes into potential proteins and searching for receptor specific protein and/or transmembrane domains. Both receptors classes are highly diverse in the slugs, and many new domain arrangements for each receptor class were found. The analyses of the gene expression of these three species provided a set of species‐specific candidate genes, that is, SR‐Bs, SR‐Es, C‐type lectins, and TSRs, that are potentially relevant for the recognition of kleptoplasts. The results set the base for future experimental studies to understand if and how these candidate receptors are indeed involved in chloroplast recognition.

## Graphical abstract

We investigated the abundance and gene expression of scavenger receptors and thrombospondin‐type‐1 repeat protein superfamily, known to be involved in symbiont recognition, in three plastid‐bearing sea slugs. We could identify candidate receptors, similar to those found in cnidarians for *Symbiodinium* recognition, that might be relevant in plastid recognition.

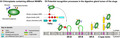

## 1 INTRODUCTION

Animals of many metazoan phyla establish a mutualistic symbiotic relationship with photosynthetic partners (Melo Clavijo et al., 2018). This so‐called photosymbiosis allows the respective host to passively gain access to the benefits of photosynthesis, while the symbionts are protected against biotic and abiotic factors and are supplied with compounds relevant for the photosynthesis, such as CO_2_ (Davy et al., 2012; Dean et al., 2016; Muscatine & Porter, 1977). Photosymbiotic processes, like the initiation of the symbiosis, mechanisms of symbiosis disruption, and the physiological benefits of both partners, are probably best understood in cnidarians (Davy et al., 2012; Fransolet et al., 2012; Koike et al., 2004; Lehnert et al., 2014; Neubauer, Poole, Detournay, Weis, & Davy, 2017; Neubauer, Poole, Neubauer, et al., 2017; Schwarz et al., 2008; van der Burg et al., 2016; Wood‐Charlson et al., 2006). However, in other photosymbiotic systems, for example, in sacoglossan sea slugs, these mechanisms are less understood. Sacoglossa sea slugs suck out the cell content of their prey, mainly macroalgae, and some species then exclusively incorporate the chloroplasts into their own cytosol (de Vries, Christa, & Gould, 2014). These “stolen plastids” (kleptoplasts) retain their photosynthetic activity even for weeks or months in the absence of any nuclear support from their original host (Händeler et al., 2009; Rauch et al., 2017; Wägele et al., 2011). This photosymbiotic system involving an animal host and photosynthetically active kleptoplasts is called “functional kleptoplasty” (Gilyarov, 1983; Waugh & Clark, 1986) and in metazoans it was only further described for two rhabdocoelan species (van Steenkiste et al., 2019).

**FIGURE 1:**
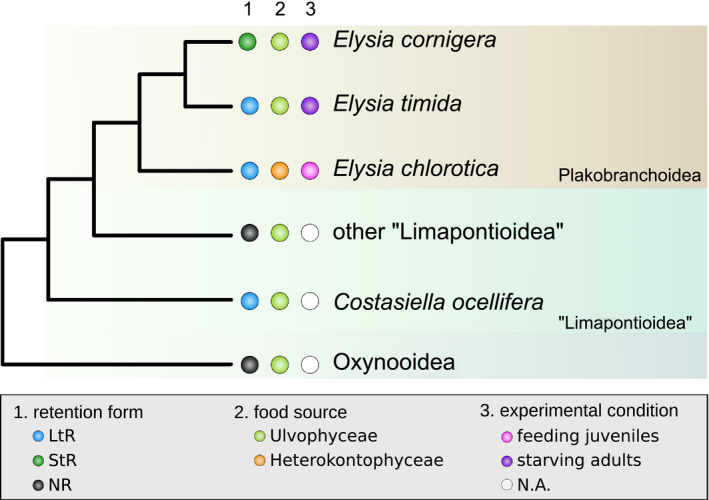
Simplified phylogenetic relationship based on Christa et al. (2015) of *Elysia timida*, *Elysia cornigera*, and *Elysia chlorotica* as well as information on the retention form and food algae, and the experimental condition the three slugs were exposed to. The shelled Oxynooidea are the most basal Sacoglossa. *Costasiella ocellifera* is the only known member of the paraphyletic “Limapontioidea” in which long‐term functional kleptoplasty is known (Christa et al., 2015)

Most Sacoglossa species are not able to retain functional kleptoplasts and even in the species that do, the stability of the association varies (see, e.g., Christa et al., 2015, 2017; Cruz et al., 2014; de Vries et al., 2015). For instance, the shelled Oxynooidea and most of the shell‐less “Limapontioidea” are not able to incorporate functional kleptoplasts (non‐retention, NR; Figure 1). However, some members of the Costasiellidae and most members of the Plakobranchoidea retain the chloroplasts for a few days up to a couple of weeks (short‐term retention, StR; Figure 1) (Christa et al., 2014; Händeler et al., 2009). Only five species are known in which the kleptoplasts are photosynthetically active for more than three months (long‐term retention, LtR) (Christa et al., 2015). Among functional plastid‐bearing Sacoglossa, the LtR species *Elysia chlorotica* Gould, 1870 and *Elysia timida* Risso, 1818, as well as the StR species *Elysia cornigera* Nuttall, 1989 are the most intensively investigated species (see, e.g., de Vries et al., 2014; Gimenez‐Casalduero et al., 2011; Rumpho et al., 2008). It has been hypothesized that in *E. chlorotica* (LtR) functional kleptoplasty takes at least seven to 10 days postmetamorphosis to become stable. Generally, the process toward a stable functional kleptoplasty can be split into an initial phase, in which the chloroplasts are primarily recognized, a transient phase, in which the kleptoplasts are incorporated but still digested, and a stable phase, in which the kleptoplasts support the slugs during development by to a small degree (Pelletreau et al., 2012).

The reasons for the different abilities to maintain functional kleptoplasty are still unknown. Based on observations that the food source alone is not sufficient (Christa et al., 2015), it is assumed that the right combination of abiotic factors, such as temperature (Laetz & Wägele, 2018), slug intrinsic factors (i.e., genomic adaptations to support the kleptoplasts), and algae chloroplast intrinsic factors (i.e., longevity of proteins relevant for photosynthesis), leads to long‐term functional kleptoplasty (de Vries et al., 2014).

Nevertheless, the food source might be important for the initiation of the symbiosis. While *E. timida* (LtR) and *E. cornigera* (StR) feed on the ulvophyte *Acetabularia acetabulum* P.C Silva, 1952 (Christa et al., 2013; de Vries et al., 2014), *E. chlorotica* (LtR) feeds on the heterokontophyte *Vaucheria litorea* C. Agardh, 1823 (Rumpho et al., 2000) (Figure 1). However, the evolutionary origin of the chloroplasts of these two algae is quite different. Chloroplasts of *A. acetabulum* evolved from a primary endosymbiosis in the chlorophyte lineage, while those of *V. litorea* evolved from a secondary endosymbiotic event in the rhodophyte lineage (Gould et al., 2008). The different evolutionary origin of the chloroplasts, and with this potential differences in the composition of glycans or lipopolysaccharides of the inner and outer chloroplast membranes, might thus have implications on their recognition by the host. However, it remains to be understood how the slugs specifically recognize the chloroplasts and if the recognition differs for kleptoplasts originating from different algal lineages.

The innate immune system probably plays a major role in the initiation of the photosymbiosis process (van der Burg et al., 2016; Davy et al., 2012; Fransolet et al., 2012; Koike et al., 2004; Lehnert et al., 2014; Mansfield & Gilmore, 2019; Neubauer, Poole, Detournay, et al., 2017; Neubauer, Poole, Neubauer, et al., 2017; Poole et al., 2016; Schwarz et al., 2008; Wood‐Charlson et al., 2006). Particularly, interactions between pattern recognition receptors (PRRs) of the host cell and microbe‐associated molecular patterns (MAMPs) of the microbe/symbiont cell trigger different signaling cascades, which are essential to discriminate a pathogen from a symbiont (Davy et al., 2012; Fransolet et al., 2012; Wood‐Charlson et al., 2006). Among PRRs, innate immune receptors, such as scavenger receptors (SRs), extracellular matrix proteins, like the thrombospondin‐type‐1 repeat (TSR) domain‐containing proteins, and cnidarian ficolin‐like receptors (CniFLs), have been shown to be involved in symbiont recognition (Baumgarten et al., 2015; Davy et al., 2012; Mansfield & Gilmore, 2019; Neubauer, Poole, Detournay, et al., 2017; Neubauer, Poole, Neubauer, et al., 2017; Rodriguez‐Lanetty et al., 2006; van der Burg et al., 2016; Wood‐Charlson et al., 2006). Especially, receptors of the SR‐B and SR‐E class play a major role in symbiont recognition (reviewed in Davy et al., 2012; Mansfield & Gilmore, 2019) and SR‐B receptors are further thought to interact with proteins containing TSR domains. This interaction might trigger the immunosuppressive transforming growth factor β (TGF‐β) pathway (Detournay et al., 2012; Li et al., 2006; Masli et al., 2006; Yehualaeshet et al., 1999), which seems to prevent a host immune response and to promote symbiont colonization and establishment (Detournay et al., 2012).

In sacoglossan sea slugs, a detailed examination of PRRs is missing and was so far only briefly investigated in the LtR species *Elysia chlorotica* (Chan et al., 2018). Here, we describe the abundance of SRs and TSRs in the StR species *E. cornigera*, and the LtR species *E. timida,* and *E. chlorotica*. To this end, we analyzed the available differential gene expression data with regard to the specific expression of both PRR groups, either during the different stages toward a stable functional kleptoplasty (*E. chlorotica*), or in freshly fed animals compared to different starvation periods (*E. cornigera* and *E. timida*). Our results revealed that Sacoglossa have a diverse SR and TSR repertoire, similar to photosymbiotic cnidarians. The expression profiles of the two PRR classes provided a set of species‐specific candidate genes that might be involved in chloroplast recognition in Sacoglossa.

## 2 METHODS

### 2.1 Analyzed species

Publicly available RNA datasets of three Sacoglossa species were used for the analyses of the abundance and expression of SRs and TSRs: *Elysia chlorotica* (LtR; NCBI SRA sample accession SRS3101883) (Chan et al., 2018), *Elysia timida* (LtR; SRS706683), and *Elysia cornigera* (StR; SRS706681) (de Vries et al., 2015). The retrieved datasets were generated under different experimental conditions: From *Elysia chlorotica*, total RNA was extracted by pooling > 20 individuals each from unfed juveniles (aposymbiotic) and from juveniles feeding for five, seven, and 10 days postmetamorphosis (Chan et al., 2018), always in triplicates. From *Elysia timida* and *Elysia cornigera*, total RNA was extracted by pooling > seven individuals from freshly fed adults (fed) and from adults starved for four and seven days, and additionally from adults starved for 30 days for *E. timida*(de Vries et al., 2015).

### 2.2 Annotation of transcriptomic data

For all species, the available assembled transcriptomes (*Elysia cornigera*: NCBI TSA version GBRW00000000.1; *E. timida*: TSA version GBRM00000000.1; *E. chlorotica:* http://cyanophora.rutgers.edu/Elysia‐expression/) were first clustered using CD‐HIT v4.6.8 with default parameters (Fu et al., 2012; Li & Godzik, 2006). For *Elysia cornigera,* we obtained 458,434 transcript clusters, for *E*. timida 274,479, and for *E. chlorotica* 129,716. The clustered transcriptomes were translated into the longest open reading frame to retrieve potential proteins using TransDecoder v3.0.1 (Haas & Papanicolaou, 2015) with default settings. The datasets were then subjected to a BLASTP search against the UniProt database version 11/13/19 (The UniProt Consortium, 2019) setting the E‐value to 1e^‐10^. Taxonomic assignment for each protein sequence was performed using the UniProt taxonomic database and sequences were subsequently filtered for Metazoa annotations (Appendix S1). Using this approach, we obtained 29,444 annotated proteins for *E. cornigera*, 20,445 for *E. timida*, and 13,389 for *E. chlorotica*.

### 2.3 Identification of scavenger receptors and thrombospondin‐type‐1 repeat proteins

The domain architecture of the filtered protein sequences was characterized by using HMMER v.3.1b2 (Eddy, Wheeler, & the HMMER Development Team, 2015) with default settings against the protein database PfamA 31.0 (Finn et al., 2016). Transmembrane regions (TM) were identified using the TMHMM server v.2.0 (Krogh et al., 2001; Sonnhammer et al., 1998). Sequences were then filtered for the different receptor class specific domains, as defined in PrabhuDas et al. (2014, 2017). For example, protein sequences having an N‐terminal cytoplasmic tail, a transmembrane domain, spacer region, α‐helical coiled coil domain, collagen domain, and a C‐terminal scavenger receptor cysteine‐rich (SRCR) domain were annotated as a member of the SR‐A class; protein sequences containing a CD36 domain in the form of an extracellular loop flanked by two transmembrane regions were annotated as a member of the SR‐B class; protein sequences having a transmembrane region with a single C‐type lectin domain were annotated as a member of the SR‐E‐like class, because sequence homology is not sufficient to include them in a SR‐E group. To be classified as SR‐E, a scavenger activity must be experimentally demonstrated (PrabhuDas et al., 2014, 2017). Protein sequences containing a transmembrane region with multiple SRCR domains were annotated as a member of the SR‐I class. All proteins that contained C‐type lectin domains, at least one transmembrane domain, and which could not be assigned to SR‐Es were classified as C‐type lectins. All proteins containing SRCR domains and that would not be assigned to SRs were classified as SRCR members.

Protein sequences were characterized as a member of the TSR superfamily if they contained a thrombospondin‐type‐1 (TSP1) domain, a disintegrin and metalloproteinase with thrombospondin motifs spacer 1 domain (ADAMTS Spacer 1), or a Sema domain. A further classification of the various TSR family members followed the definition given by Adams and Tucker (2000), Tucker (2004), and Adams and Lawler (2011). For instance, thrombospondins (TSPs) have an invariant carboxy‐terminal region consisting of repeats of epidermal growth factor (EGF)‐like domains, 13 calcium‐binding type 3 repeats, a homologous L‐type lectin domain in the C‐terminal region, and N‐terminal region that varies in domain composition (Adams & Lawler, 2011). Repeats of the TSP1 domain are named as TSR. The TSR domain in cnidarians is similar to that in vertebrates (Adams & Tucker, 2000; Silverstein, 2002; Tan et al., 2002). It includes six cysteine residues, a protein and glycosaminoglycan (GAG) binding site formed by the motif WXXWXXW, a RXRXRX motif consisting of polar residues (such as arginine, lysine, and glutamine). Further, it contains binding regions for SR‐B proteins formed by the motifs CSVTCG and GVQTRXR (Neubauer, Poole, Neubauer, et al., 2017). Members of the ADAMTS group have a signal peptide, a prodomain, a metalloproteinase catalytic domain, a disintegrin‐like domain, a central TSP1‐like domain repeat, a cysteine‐rich domain, a spacer region with variable length, and C‐terminus with a variable number of TSP1 domains (Porter et al., 2005). Semaphorins, a group of secreted and transmembrane proteins, were identified by the presence of the Sema domain (Raper, 2000). Out of the eight classes of semaphorins (1 to 7 plus class V for viruses), class 5 is also classified as TSR, due to the presence of the TSP1 domain (Adams & Tucker, 2000; Tucker, 2004). Properdin, a further member of the TSR superfamily, is characterized only by the presence of six consecutive TSP1 domains (Nolan et al., 1991, 1992; Sun et al., 2004). In the present study, sequences similar to properdin were defined as TSR‐TM (without transmembrane regions). Sequences containing only TSP1 domains with a transmembrane region were grouped as TSR + TM. Proteins were further filtered for a minimum length of 150 amino acids and an independent E‐value of 1e^‐5^ as recommended in the manual of HMMER v.3.1b2 (Eddy et al., 2015). A sequence logo of the TSP1 domains of those TSR sequences that were differentially expressed (see below) was created using the weblogo server (http://weblogo.berkeley.edu/logo.cgi) and compared to the general Pfam TSP1 domain motif downloaded from https://pfam.xfam.org/.

### 2.4 Gene expression analyses

The datasets used in this study were previously analyzed using different tools. In order to avoid any method‐based difference, we de novo analyzed the gene expression. For this, the respective short reads were downloaded for each species from the short read archive deposited in GenBank (see above) analyses. Reads were then mapped using Bowtie2 v2.3.4.3 (Langmead & Salzberg, 2012) onto the clustered transcriptomes. Transcript abundance of sequences with a raw read count of at least 100 raw counts in any two samples tested was estimated using RSEM (Li & Dewey, 2011) implemented in Trinity v.2.9.0 (Grabherr et al., 2011). Differential gene expression analyses were performed using edgeR v3.30.3 (Robinson et al., 2010). For feeding juveniles of *E. chlorotica*, we compared specimens fed for five days to the aposymbiotic state (initial); specimens fed for seven days with specimens fed for five days (transient); and specimens fed for 10 days with specimens fed for seven days (stable), to investigate whether the expression of the receptors changed during the different hypothesized stages to establish a stable functional kleptoplasty (Pelletreau et al., 2012). For *E. timida* and *E. cornigera,* we compared the freshly fed animals to the different starvation periods, in order to identify genes that might be relevant, while the slugs are feeding. We then focused on genes that were highly expressed in feeding animals compared to all starvation periods. Only genes with a log_2_ fold change (L2FC) >1 or <−1 were considered as significantly differentially expressed, because we assumed the expression of a gene to be relevant when it changes twofold. Further, because for *E. cornigera* and *E. timida* no biological replicates are available, we used a L2FC threshold of < −1 or >1 as a way to infer meaningful expression changes.

## 3 RESULTS

### 3.1 Abundance of scavenger receptors in *Elysias*

**FIGURE 2:**
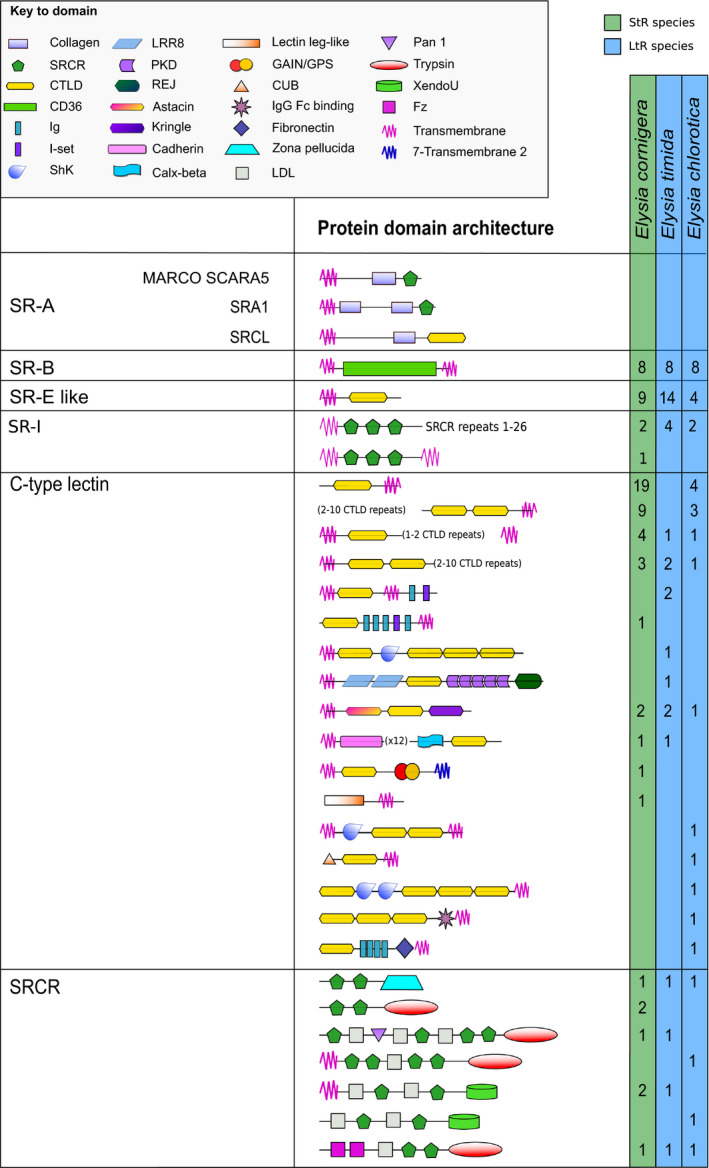
Overview about the diversity of scavenger receptor proteins in *Elysia timida*, *Elysia cornigera,* and *Elysia chlorotica*. As no putative SR‐A proteins were identified in any of the slugs, the general domain architecture of SR‐A proteins in humans is shown as a reference. Astacin, peptidase family M12A; CD36, cluster of differentiation 36; CTLD, C‐type lectin domain; GAIN/GPS, G protein‐coupled receptors autoproteolysis inducing domain; Ig, immunoglobulin domain; I‐set, immunoglobulin I‐set domain; LDL, low‐density lipoprotein domain; LRR8, leucine‐rich repeat 8 domain; PKD, polycystic kidney disease domain; REJ, receptor of egg jelly domain; ShK, *Stichodactyla helianthus* K^+^ channel toxin domain; SRCR, scavenger receptor cysteine‐rich domain

No putative SR‐A receptor proteins could be identified in any of the investigated *Elysia* species. A total of eight potential SR‐B proteins, a varying number of SR‐E‐like proteins, with the highest number identified in *E. timida* (15), and two to four SR‐I proteins were found in all sea slugs (Figure 2). Additionally, numerous protein sequences containing one or multiple CTLD, often combined with various other domains, were found. Especially in *E. cornigera,* a high diversity (41) of C‐type lectin proteins was found. Additionally, proteins containing SRCR domains combined with other domains were found in all three slugs (Figure 2).

### 3.2 Abundance of TSRs in *Elysias*

**FIGURE 3:**
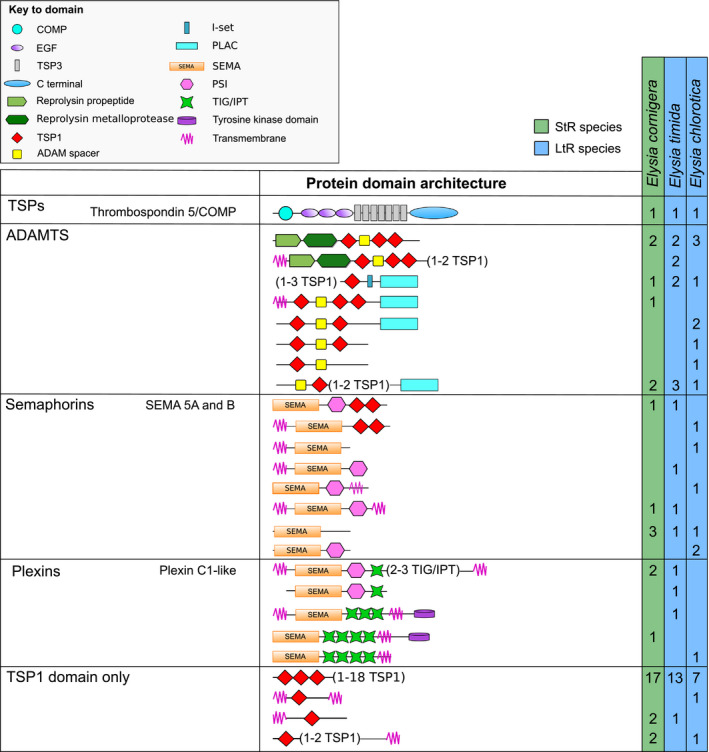
Overview about the diversity of thrombospondin‐type‐1 repeat (TSR) proteins in *Elysia timida*, *Elysia cornigera,* and *Elysia chlorotica*. COMP, cartilage oligomeric matrix protein; EGF, epidermal growth factor domain; I‐set, immunoglobulin I‐set domain; PLAC, protease and lacunin domain; PSI, plexin repeat; TIG/IPT, immunoglobulin‐like, plexins, transcription factor domain; TSP1, thrombospondin‐type‐1 domain; TSP3, thrombospondin‐type‐3 domain

**FIGURE 4:**
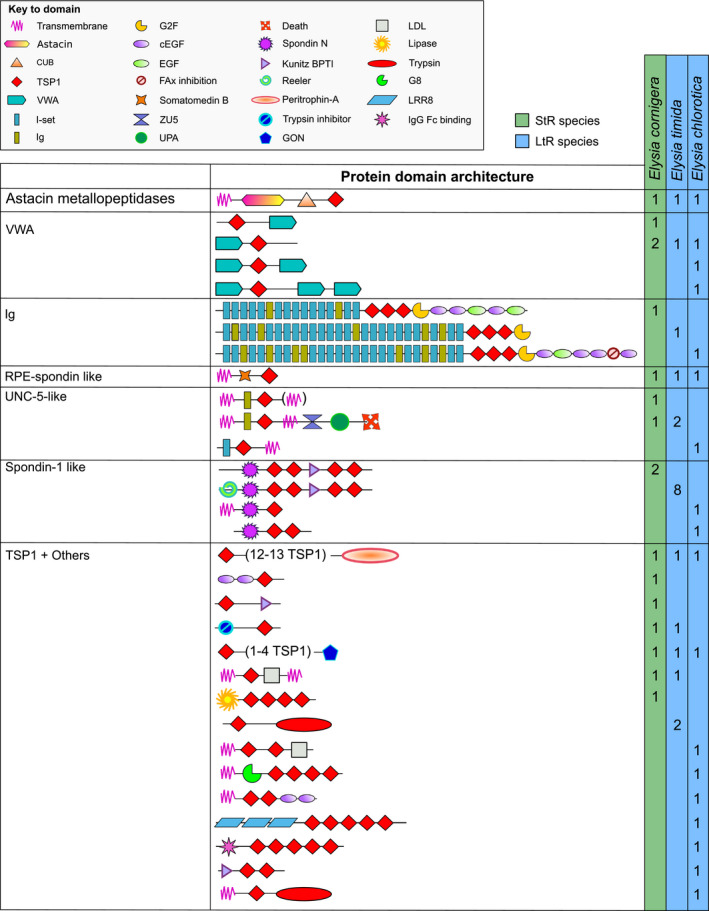
Overview about the diversity of new domain arrangements of thrombospondin–type‐1 repeat (TSR) proteins in *Elysia timida*, *Elysia cornigera*, and *Elysia chlorotica*. CUB, complement C1r/C1s, Uegf, Bmp1 domain; EGF, epidermal growth factor domain; Ig, immunoglobulin domain; I‐set, immunoglobulin I‐set domain; Kunitz BPTI, Kunitz bovine pancreatic trypsin inhibitor domain; LDL, low‐density lipoprotein domain; LRR8, leucine‐rich repeat 8 domain; TSP1, thrombospondin‐type‐1 domain; VWA, von Willebrand factor type A domain

In all analyzed *Elysia* species, a potential thrombospondin type 5/cartilage oligomeric matrix protein (COMP) homologue was identified (Figure 3). Additionally, several different putative ADAMTS‐like, semaphorin, and plexin homologues were found. The vast majority of TSR sequences only contained TSP1 sequences (Figure 3). Furthermore, for each species an Astacin homologue and several homologues containing either of von Willebrand factor A (VWA) domains or immunoglobulin‐like domains, or UNC‐5 domains, as well as putative homologues of RPE‐spondin and spondin (Figure 4). The highest diversity of TSR receptors (15 different arrangements) was found for proteins that contained one or several TSP1 domains combined with a variety of different receptors (Figure 4).

### 3.3 Expression of PRRs in adults of the StR *E. cornigera* and the LtR *E. timida*

**FIGURE 5:**
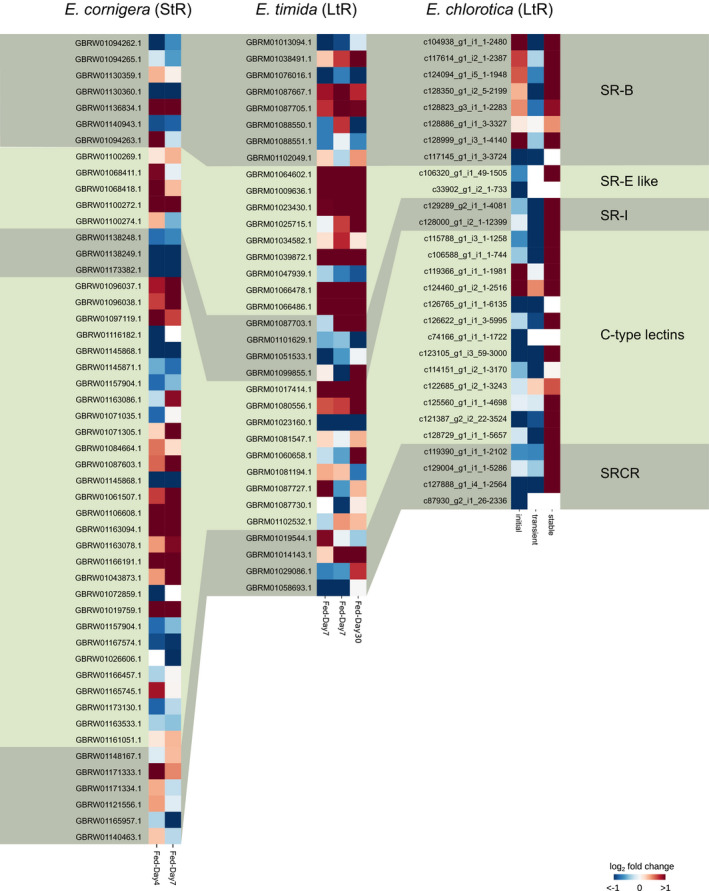
Gene expression profile of scavenger receptors in *Elysia cornigera* (StR)*, Elysia timida* (LtR) and *Elysia chlorotica* (LtR). Shown is the log_2_ fold change (L2FC) of the gene expression and only genes that were differentially expressed in at least one condition are displayed

In the StR species *E. cornigera*, six out of 68 genes belonging to the SRs class were significantly upregulated in the freshly fed animal, compared to both starvation periods (Table 1, Figure 5). One of these genes is a putative SR‐B homologue (GBRW01136834.1), a putative Perlucin homologue (GBRW01100272.1) belonging to the SR‐E receptors, and four C‐type lectins. Among the C‐type lectins are two putative C‐type mannose receptor 2 sequence homologues (GBRW01106608.1 and GBRW01163094.1), and one putative Versican core protein homologue (GBRW01163094.1) that all contained two C‐type lectin domains (CTLD) and one transmembrane (TM) region. Further, we found one gene to be significantly upregulated (GBRW01019759.1) which contained two CTLDs flanked at both sides by a TM region and was annotated as putative Snaclec agglucetin subunit beta‐1 homologue.

**TABLE 1:** Scavenger receptors and thrombospondin‐type 1 receptors of the StR species *Elysia cornigera* and the LtR species *Elysia timida* that were significantly upregulated in freshly fed animals

Species	Receptor	Domain arrangement	Gene_ID	UniProtKB annotation	log_2_ fold change		
					Day 4	Day 7	Day 30
*Elysia cornigera* (StR)	SR‐B	TM + CD36 + TM	GBRW01136834.1	Scavenger receptor class B member 1	2.41	1.43	–
	SR‐E like	TM + CTLD	GBRW01100272.1	Perlucin	3.04	1.32	–
	C‐type lectins	CTLD + CTLD +TM	GBRW01106608.1	C‐type mannose receptor 2	2.53	8.50	–
			GBRW01166191.1	C‐type mannose receptor 2	2.11	1.15	–
			GBRW01163094.1	Versican core protein	1.22	2.28	–
		TM + CTLD +CTLD + TM	GBRW01019759.1	Snaclec agglucetin subunit beta‐1	1.69	1.17	–
	TSP1	TSP1	GBRW01123401.1	Hemicentin‐1	3.24	2.69	–
*Elysia timida* (LtR)	SR‐E like	TM + CTLD	GBRM01064602.1	Collectin‐10	2.66	3.54	2.08
			GBRM01009636.1	C‐type lectin 37 Da	2.80	2.54	6.14
			GBRM01066486.1	C‐type lectin 37 Da	3.87	3.66	6.09
			GBRM01066478.1	Perlucin	3.69	3.92	4.63
			GBRM01039872.1	C‐type mannose receptor 2	2.90	2.61	2.36
	C‐type lectins	TM + CTLD +TM	GBRM01017414.1	Collectin‐12	3.58	1.73	2.72
	ADAMTS	TSP1 + TSP1 + I‐set + Plac	GBRM01039431.1	Protein madd‐4	8.92	9.47	10.06

Note

The log_2_ fold change shows the expression in freshly fed animals compared to the respective starvation period.

Abbreviations: CD36, cluster of differentiation 36; CTLD, C‐type lectin domain; I‐set, immunoglobulin I‐set domain; Plac, protease and lacunin domain; TM, transmembrane region; TSP1, thrombospondin‐type‐1 domain.

**FIGURE 6:**
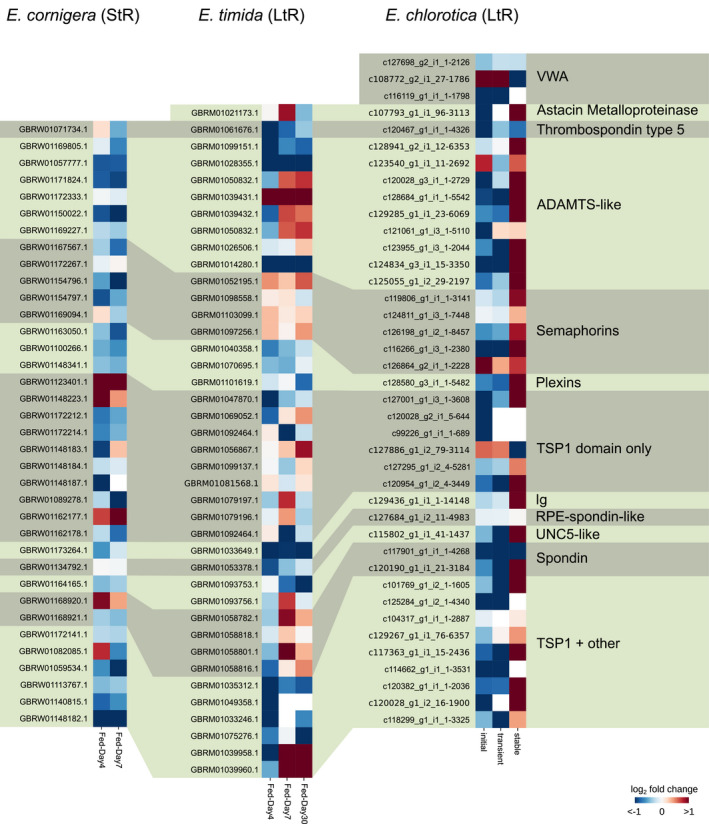
Gene expression profile of thrombospondin‐type‐1 repeat (TSR) proteins in *Elysia cornigera* (StR)*, Elysia timida* (LtR) and *Elysia chlorotica* (LtR). Shown is the log_2_ fold change (L2FC) of the gene expression and only genes that were differentially expressed in at least one condition are displayed

In freshly fed animals of the StR *E. cornigera,* only one out of 53 identified TSRs was significantly expressed compared to both starvation conditions (Table 1, Figure 6). This sequence (GBRW01123401.1) only contained one TSP domain and was annotated as Hemicentin‐1.

Regardless of the starvation duration, six out of the 40 genes classified as SRs were significantly upregulated in the freshly fed animal of the LtR species *Elysia timida* (Table 1, Figure 5). Five of those genes belonged to the SR‐E like class, all containing one TM and one CTLD. Among those genes were a putative Collectin‐10 (GBRM01064602.1), two C‐type lectin 37Da homologues (GBRM01009636.1 and GBRM01066486.1), one putative Perlucin homologue (GBRM01066478.1), and one putative C‐type mannose receptor 2 homologue (GBRM01039872.1). Additionally, one C‐type lectin homologue (GBRM01017414.1), containing one CTLD flanked at both sites by TM regions, was significantly upregulated.

In freshly fed adults of the LtR *E. timida,* only one out of 52 TSR genes was significantly upregulated compared to all three starvation conditions (Table 1, Figure 6). This gene (GBRM01039431.1) was classified as ADAMTS member and contained two TSP domains, one I‐set domain, and one PLAC domain and was annotated as Protein madd‐4 homologue.

### 3.4 Expression of PRRs in feeding juveniles of the LtR *E. chlorotica*

In feeding juveniles of the LtR species *Elysia chlorotica,* four of the 33 receptors classified as SRs were expressed during the initial phase of functional kleptoplasty (Table 2, Figure 5). Out of those two SR‐B receptor homologues (c104938_g1_i1_1‐2480 and c128999_g1_i3_1‐4140) and two genes belonging to the C‐type lectins, a putative snaclec B1 homologue (c119366_g1_i1_1‐1981) and a putative secretory phospholipase A2 receptor homologue (c124460_g1_i2_1‐2516) were significantly upregulated during the initial phase of functional kleptoplasty (Figure 5). During the transient phase of kleptoplasty, one of the previous upregulated SR‐B homologues (c104938_g1_i1_1‐2480) was significantly down‐regulated, while the other SR‐B homologue and the two C‐type lectins were not significantly altered. During the stable phase, the gene expression of SRs changed considerably. Out of 33 genes, 20 were significantly upregulated, while the remaining sequences did not change significantly. Overall, five SR‐B, one SR‐E like, both SR‐I, nine C‐type lectins, and three SRCR homologues were upregulated during the stable phase.

**TABLE 2:** Scavenger receptors and thrombospondin‐type‐1 receptors of the LtR species *Elysia chlorotica*

Species	Receptor	Domain arrangement	Gene_ID	UniportKB annotation	log_2_ fold change		
					Initial	Transient	Stable
*Elysia chlorotica* (LtR)	SR‐B	TM + CD36 + TM	c104938_g1_i1_1‐2480	Lysosome membrane protein 2	2.02	−2.12	1.33
			c128999_g1_i3_1‐4140	Scavenger receptor class B member 1	2.58	−0.38	1.90
	C‐type lectins	CTLD + CTLD +TM	c119366_g1_i1_1‐1981	Snaclec B1	1.10	−0.05	3.17
			c124460_g1_i2_1‐2516	Secretory phospholipase A2 receptor	1.68	0.48	2.95
	VWA	VWA + TSP1 + VWA	c108772_g2_i1_27‐1786	Collagen alpha−5 chain	1.05	2.00	−1.55
	Semaphorin	SEMA + PSI	c126864_g2_i1_1‐2228	Plexin‐B	1.33	0.39	0.72

Note

The log_2_ fold change shows the expression during the different stages of functional kleptoplasty. The focus was set on genes expressed during the initial phase.

Abbreviations: CD36, cluster of differentiation 36; CTLD, C‐type lectin domain; SEMA, Sema domain; TM, transmembrane region;TSP1, thrombospondin‐type‐1 domain; VWA, von Willebrand factor type A domain.

In feeding juveniles of the LtR species *E. chlorotica,* only two TSR genes, a collagen alpha‐5 chain homologue (c126864_g2_i1_1‐2228), which contains a VWA and a TSP1 domain, as well as a Plexin‐B homologue (c126864_g2_i1_1‐2228), which contains a Sema domain (SEMA) and a PSI integrin domain (PSI), were significantly upregulated during the initial phase of functional kleptoplasty (Table 2, Figure 6). This general expression profile does not change during the transient phase of functional kleptoplasty, with the exception that the Plexin‐B homologue (L2FC 0.39) was not significantly regulated anymore. During the stable phase of functional kleptoplasty, the expression profile of TSRs changes, similar as of SRs, extensively (Figure 6). Overall, 18 genes were significantly upregulated during the stable phase, three significantly down‐regulated, and 19 did not change. Especially, seven ADAMTS domain‐containing proteins were significantly upregulated (average L2FC 2.13 ± 0.67). Among genes containing only TSP1 domains, two were significantly upregulated as well; a Hemicentin‐1 homologue (c127001_g1_i3_1‐3608, L2FC 1.04) containing 14 TSP1 domains and a thrombospondin‐type‐1 domain‐containing protein 7A homologue (c120954_g1_i2_4‐3449, L2FC 1.48) containing two TSP1 domains and a transmembrane region.

**FIGURE 7:**
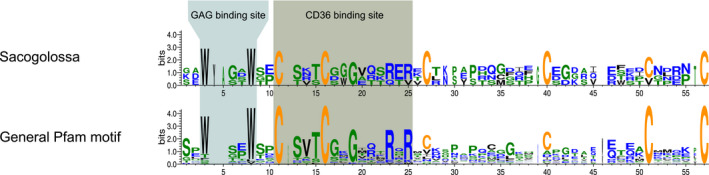
Sequence logo of the TSP1 domains of TSR genes that were differentially expressed throughout the experimental conditions in the three slugs. The profile was compared to the general Pfam profile of TSP1 domains. The glycosaminoglycan (GAG) and CD36 binding sites are highlighted in the boxes, and the six conserved cysteines are shown in orange

Generally, the TSP1 domains of the upregulated TSR sequences of the slugs were made out of six conserved cystein residues, containing a WXXW (where X is any amino acid) motif, a motif similar to the CSVTCG motif and a subsequent RXR motif (Figure 7).

## 4 DISCUSSION

To shed light on the initiation of functional kleptoplasty, we analyzed the abundance and domain architecture of scavenger receptors (SRs) and thrombospondin‐type‐1 repeat (TSR) protein superfamily in three kleptoplastid‐bearing sea slugs *Elysia cornigera* (StR), *E. timida* (LtR), and *E. chlorotica* (LtR). All species possess a similar SRs and TSRs receptor repertoire independent on the ability to either maintain the kleptoplasts in the short or long term. We could only find minor differences in the number of genes and the diversity of some receptors mainly between *E. cornigera*/*E. timida* and *E. chlorotica*. This might, however, be based on the different experimental setups and developmental stage of the used specimens with an according different gene expression profile, rather than genomic differences, for example, gene duplication, diversification, or losses. Nevertheless, the general abundance is also similar to that found in cnidarians (Neubauer, Poole, Detournay, et al., 2017; Neubauer, Poole, Neubauer, et al., 2017).

**FIGURE 8:**
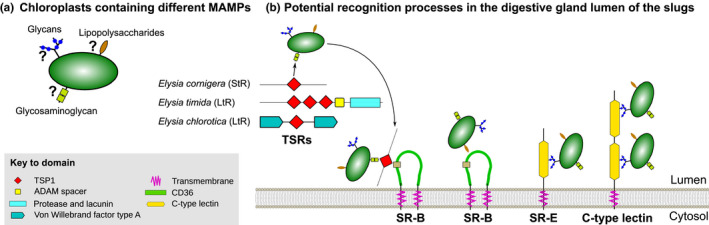
Schematic overview of potential recognition processes in Sacoglossa sea slugs. (a) The exact composition of glycans, lipopolysaccharides, and glycosaminoglycans of the chloroplast is still unknown for *Acetabularia acetabulum* and *Vaucheria litorea*. (b) TSRs are expressed in a species‐specific manner and might bind to glycosaminoglycan to enhance binding to SR‐B. The chloroplasts can potentially also directly bind to SR‐Bs through lipopolysaccharides. Further, SR‐E and C‐type lectins can bind to glycans

Independently of the experimental condition, the gene expression profiles of the three species provided a set of species‐specific candidate genes, in particular SR‐B, SR‐E, C‐type lectins, and TSR genes, that might be relevant for plastid recognition (Figure 8). Receptors belonging to those classes are likewise upregulated during the onset of a symbiosis in cnidarians (Mohamed et al., 2016; Neubauer, Poole, Neubauer, et al., 2017) and at least for SR‐Bs and TSRs their involvement in symbiont recognition was verified by physiological trials (Neubauer, Poole, Detournay, et al., 2017; Neubauer, Poole, Neubauer, et al., 2017). We found TSP1 domain motifs of the TSR proteins (six conserved cystein residues, the protein and glycosaminoglycan binding motif, and a motif similar to the CD36 binding motif) (Zhang & Lawler, 2007), that are needed for potential interactions of the TSRs with SR‐B receptors (Detournay et al., 2012; Neubauer, Poole, Neubauer, et al., 2017). The candidate TSR genes identified in the slugs differ among the three species and to those known from cnidarians, where a previous analysis of the gene expression showed an upregulation of semaphorin 5A and a trypsin‐like gene (Neubauer, Poole, Neubauer, et al., 2017). Thus, if there is an interaction of TSRs with SR‐Bs in the slugs, the exact TSR proteins involved vary with the different slugs and compared to cnidarians. Yet, whether the identified candidate genes in the slugs are indeed involved in symbiont recognition and whether the TSRs are interacting with the SR‐Bs (Figure 8), and hence are involved in promoting the symbiont tolerance by the host (Detournay et al., 2012; Mansfield & Gilmore, 2019), remains to be experimentally tested.

CTLD‐containing receptors are upregulated in all three slugs, and evidence exists that glycan–lectin interactions are relevant in the symbiont recognition process in cnidarians (reviewed in Davy et al., 2012; Mansfield & Gilmore, 2019). For instance, lectins can bind to conserved glycans in *Symbiodinium* cell walls inducing phagocytic processes, and the activation of the complement pathway (Davy et al., 2012; Fransolet et al., 2012; Koike et al., 2004; Lin et al., 2000; Logan et al., 2010; Poole et al., 2016; Wood‐Charlson et al., 2006). Lectins have been found surrounding symbionts in gastrodermal host cells (Jimbo et al., 2000, 2005; Kvennefors et al., 2008, 2010) and they can induce *Symbiodinium* transformation, from the motile stage to a coccoid nonmotile stage, suitable for the symbiosis establishment (Koike et al., 2004).

An involvement of lectins in chloroplast recognition is, however, uncertain. In some plant species, for instance in the pea *Pisum sativum* (Keegstra & Cline, 1982), the outer membrane of the chloroplasts lacks glycoproteins, which would prevent interactions with lectins. Whether the chloroplasts of the Sacoglossan food sources lack glycoproteins too is unknown (Figure 8). The outer envelope of primary chloroplasts, as in *Acetabularia acetabulum*, the food source of *E. cornigera* and *E. timida*, is generally rich in galactolipids (mono‐ and digalactosyldiacylglycerol), phosphatidylcholine, and low in phospholipids, with a small portion of phosphatidylglycerol (Block et al., 2007; Keegstra & Yousif, 1986), and has the highest lipid to protein ratio among any plant membrane (Block et al., 1983). Secondary chloroplasts, as in *Vaucheria litorea*, the food source of *E. chlorotica*, possess three to four membranes consisting also of mono‐ and digalactosyldiacylglycerol, although the precise location of these galactolipids in the various plastid membranes is still unclear (Petroutsos et al., 2014). In the case of *V. litorea* plastids, the membranes are also associated with the endoplasmic reticulum in what is called the chloroplast endoplasmic reticulum (Graves et al., 1979; Rumpho et al., 2000). In *E. chlorotica*, the outer two membranes of the kleptoplasts are, however, degraded (Rumpho et al., 2000). What mechanism underpins this degradation and whether it occurs before or after the ingestion is unknown, but might be an, additional, important factor regarding chloroplast recognition. Nevertheless, an involvement of SR‐E‐like or other C‐type lectin domain‐containing receptors should not be ruled out (Figure 8).

The recognition process also includes the release of compounds by the symbiont. For instance, glycoconjugates are thought to serve as species‐specific signaling molecules, important during recognition and maintenance of the symbiont (Markell & Wood‐Charlson, 2010). So far, there is no evidence of the secretion of potential recognition signal molecules from the chloroplast in a kleptoplastic system and at least the lipidome does not undergo any shifts during the onset of functional kleptoplasty (Rey et al., 2017). Thus, if and how the chloroplasts might be actively enhance the recognition process remains elusive.

The present study made a step toward compiling a list of candidate genes potentially involved in chloroplast recognition in Sacoglossa, but the exact mechanisms are still far from being understood. This is in part due to the fact that available transcriptomic data are heterogeneous, making it hard to infer a general pattern. Furthermore, in particular for juveniles of *E. chlorotica* it is nearly impossible to discriminate between gene expression related to development or chloroplast recognition. Based on the expression analyses between the different phases of functional kleptoplasty, it seems that during each transition, the gene expression changes considerably, especially during the stable phase of functional kleptoplasty, which could be more related to development than to functional kleptoplasty (see also Chan et al., 2018). Future research should thus focus on generating homogenous datasets including aposymbiotic animals in order to help understand how Sacoglossa can recognize and subsequently maintain their kleptoplasts. This task is, however, particularly complicated, because only for the StR species *Elysia viridis* aposymbiotic adults could be cultured so far under laboratory conditions (Rauch et al., 2018), but there is no transcriptomic dataset available for this species. Further, a comparative analysis using shelled species, that digest the chloroplast extracellularly, combined with homogenous datasets of StR and LtR species, would have the potential to allow for a better understanding of the mechanisms and the evolution of gene expression related to incorporate chloroplasts in plastid‐bearing sea slugs.

## CONFLICT OF INTEREST

The authors declare no conflict of interest.

## AUTHOR CONTRIBUTION

**Jenny Melo Clavijo:** Conceptualization (equal); Formal analysis (equal); Investigation (lead); Methodology (equal); Visualization (equal); Writing‐original draft (lead). **Silja Frankenbach:** Formal analysis (equal); Resources (equal); Validation (equal); Writing‐review & editing (equal). **Cátia Fidalgo:** Formal analysis (equal); Validation (equal); Writing‐review & editing (equal). **Joao Serôdio:** Formal analysis (equal); Resources (equal); Writing‐review & editing (equal). **Alexander Donath:** Data curation (equal); Formal analysis (equal); Methodology (equal); Validation (equal); Writing‐review & editing (equal). **Angelika Preisfeld:** Formal analysis (equal); Resources (equal); Supervision (lead); Writing‐review & editing (equal). **Gregor Christa:** Conceptualization (lead); Formal analysis (lead); Funding acquisition (lead); Investigation (lead); Methodology (equal); Project administration (lead); Resources (equal); Supervision (supporting); Visualization (lead); Writing‐original draft (lead); Writing‐review & editing (equal).

## Supporting information

Appendix S1

Appendix S2

## Data Availability

Raw reads of *Elysia chlorotica* (SRA sample accession SRS3101883), *Elysia timida* (SRS706683), and *Elysia cornigera* (SRS706681) are accessible via download from GenBank. The assembled transcriptomes are accessible via download from GenBank (*E. cornigera*: GBRW00000000.1; *E. timida*: GBRM00000000.1) or elsewhere (*E. chlorotica:* http://cyanophora.rutgers.edu/Elysia‐expression/). Annotation tables of each species can be downloaded from DRYAD: https://doi.org/10.5061/dryad.ttdz08kw2.
